# I see you, you see me: the impact of social presence on social interaction processes in autistic and non-autistic people

**DOI:** 10.1098/rstb.2021.0479

**Published:** 2023-04-24

**Authors:** Megan Freeth, Emma J. Morgan

**Affiliations:** Department of Psychology, The University of Sheffield, Sheffield, Sheffield S1 2LT, UK

**Keywords:** autism, social interaction, social communication, ecological validity, social presence

## Abstract

Environments that require social interaction are complex, challenging and sometimes experienced as overwhelming by autistic people. However, all too often theories relating to social interaction processes are created, and interventions are proposed, on the basis of data collected from studies that do not involve genuine social encounters nor do they consider the perception of social presence to be a potentially influential factor. In this review, we begin by considering why face-to-face interaction research is important in this field. We then discuss how the perception of social agency and social presence can influence conclusions about social interaction processes. We then outline some insights gained from face-to-face interaction research conducted with both autistic and non-autistic people. We finish by considering the impact of social presence on cognitive processes more broadly, including theory of mind. Overall, we demonstrate that choice of stimuli in studies assessing social interaction processes has the potential to substantially alter conclusions drawn. Ecological validity matters and social presence, in particular, is a critical factor that fundamentally impacts social interaction processes in both autistic and non-autistic people.

This article is part of a discussion meeting issue ‘Face2face: advancing the science of social interaction’.

## 1. Introduction

‘A flat-white to take out please’. ‘Be with you in a second’. Even a simple, short social interaction requires highly complex cognitive processing of multiple verbal and non-verbal cues in order to be completed successfully. No further verbal communication is necessarily required in this initial sequence for the interaction to be considered successful by both parties. This requires knowledge and understanding of the normal sequence of events, unspoken social rules and non-verbal cues in order to satisfy each party that the other is holding up their end of the social interaction contract. In this case, the outcome is likely to be that the customer will quietly wait up to a few minutes (rather than the requested ‘second’) and then expect to have a coffee delivered to their hand by the barista before making a payment. For a more involved social interaction, for example, a conversation involving the exchange of views, many separable elements must be processed simultaneously to follow the gist of the exchange. Complex information must be processed and interpreted rapidly. Listeners and speakers must take others' perspectives into account as new information emerges on a moment-by-moment basis. The thoughts and beliefs of the social partner must be considered. Verbal and non-verbal cues must be integrated in order for the intended meaning to be conveyed and interpreted. All this happens while those involved must filter out other visual and auditory noise from the environment in order to process the meaning of the social interaction. It is, therefore, no wonder that social interactions are experienced as being extremely challenging by some.

Individuals with a diagnosis on the autism spectrum experience difficulties in social communication and social interactions across multiple contexts, including difficulties in expressing and interpreting non-verbal communicative behaviours (such as eye contact and body language) according to the clinical definition (DSM-5, [1]). A huge volume of research has been conducted on this issue (see [2–5] for recent reviews) including a focus on whether social attention patterns can be used as a biomarker for autism diagnosis [6–8]. Indeed, differences between autistic and non-autistic people's attention to direct gaze have been found to exist across cultures, with a recent meta-analysis including over 2000 autistic participants demonstrating gaze atypicalities for individuals from both Eastern and Western cultures [9]. However, while many of the difficulties experienced by autistic individuals are social in nature, much previous research in this area has been conducted in laboratory environments lacking the presence of a social partner. This raises the very real concern that results found in these studies may not extrapolate to everyday behaviour [10–12]. This is an important issue, as laboratory-based studies often inform theories and interventions developed with a view to assisting people with a diagnosis on the autism spectrum. Such interventions have been found to be variable in their success and have relatively small effects [13] hence there is a clear need to do better in order to reach the goal of research being genuinely beneficial to autistic people.

The aim of this review is to consider the nature and impact of a range of socially relevant factors. These include ‘social agency’, i.e. actions or potential for actions that are derived from a social being. Further, we will consider how the sense of being present with another being that possesses social agency or that represents a social entity, i.e. ‘social presence’, affects another person's attention, information processing and social interaction behaviour. These factors will be considered in relation to both autistic and non-autistic people. Different methodologies for assessing the impact of social presence will be considered. How these processes differ, or do not differ, between autistic and non-autistic people will be discussed. We will initially consider why face-to-face social interaction research, where both the participant and their social partner are present in the same room, is important. We will then consider the contribution of a range of research whereby the introduction of a social presence is done merely by altering participants' perceptions of what they are seeing and what they are being asked to do. We will then move to discuss how to analyse the processes involved in interactivity, whereby a participant engages in research that features encounters with stimuli that possess social agency and consider the importance of how naturalistic, or close to real life experiences, an encounter is. We will then explore a range of different methodologies and paradigms that have been used to assess face-to-face encounters. We will finish by considering how face-to-face research can further understanding of cognitive mechanisms relating to social interactions that go beyond social attention, including theory of mind processes. Via this review, our aim is to provide a summary of methodological approaches to reveal the mechanisms and processes potentially influenced by a social presence in relation to the social encounter, including social interactions, with a specific focus on improving understanding of these in relation to autistic social interactions.

## 2. Why do face-to-face social interaction research?

Before we consider *how* face-to-face social interaction research can be done, it is important to understand *why* we would want to do this in the first place. Doing research where behaviour is assessed in naturalistic contexts is challenging. The real world is highly variable and unpredictable. This variation, or ‘noise’ makes it more challenging to uncover the key elements, or ‘signal’, that may be operating differently between two sets of individuals. Computer-based studies where stimuli are tightly controlled reduce the possibility of noise drowning out the signal. However, if we ignore evidence from real world paradigms when building our theoretical models, these models will inevitably be incomplete. A range of other reviews have explored this point further in neurotypical individuals, e.g. Risko *et al*. [14] and Gobel *et al*. [15]. The process of conducting research that involves a genuine social presence within paradigms has been termed ‘breaking the fourth wall’, Risko *et al*. [16]. The ‘fourth wall’ is often experienced in theatre settings whereby those present experience an illusory barrier between the actors and the audience. This allows the audience to believe that the stage is a world apart from theirs and allows the actors to overlook the fact that others are observing them. As will be demonstrated later in this review article, when stimuli in experimental paradigms can ‘look back’ at the participant, i.e. the stimulus has the potential to observe or judge, or are even simply perceived as having the potential to, commonly observed phenomena and effects can be turned on their head. For example, robustly observed effects such as the gaze cueing effect, or preferential attendance to eyes in neurotypical individuals, can significantly alter when participants believe a stimulus represents a real social partner [17,18]. This has led to fundamentally new insights and directions for research. It is therefore vital that real world studies are conducted in order to provide greater insight into social interaction processes.

## 3. The perception of social presence

An important starting point is to identify the characteristics a stimulus must possess in order to be considered ‘social’. Previous research with neurotypical individuals has indicated that, rather surprisingly, it is not always the physical characteristics of a cue that enable us to process it in a social manner, but rather our perception of a stimulus as possessing social agency, or representing a social entity. This perception can then lead to a stimulus acting as a ‘social presence’, instilling a participant with an awareness of the presence of a social partner [19]. There are numerous studies which have demonstrated the importance of a participant's perception of a stimulus, and we will discuss a select few which demonstrate this claim throughout this section. In an eminent study, Ristic & Kingstone [20] presented participants with an ambiguous stimulus that could be viewed as either a car or a face, where two large white circles containing smaller black circles were either interpreted to represent car wheels or a pair of eyes. They found that when the stimulus was referred to as a ‘face’ participants exhibited the gaze cueing effect, i.e. rapidly attending to the cued location regardless of whether or not this was beneficial to the task at hand. However, this effect was not present when the stimulus was referred to as a ‘car’. Similarly, Wiese *et al*. [17] showed participants images of both human and robot faces. Participants were informed that the eye movements of the face could be controlled by either a human or a robot. They found that gaze cueing effects were significantly larger when participants believed the faces were controlled by a human agent regardless of whether they viewed a human or robot face. Further, in a more recent study, Gobel *et al*. [19] demonstrated that participants synchronized their own eye movements to the movements of a red dot when they believed it to represent the eye movements of another participant compared to when they believed the movement was generated by a computer. Taken together, the results of these studies suggest that participants are sensitive to whether a stimulus represents a social entity or not, be this via social agency or representation of the physical manifestation of a social entity depicted as eyes, and this guides visual attention.

It has been argued that autistic people are less sensitive to the presence of other people. Indeed, much previous research has indicated that individuals with an autism diagnosis do not demonstrate a social facilitation effect [21–23], the social facilitation effect being improved performance when in the presence of others. The ‘social motivation hypothesis' proposes that autistic people have less interest in social phenomena than neurotypical people do, and therefore their behaviour is less likely to be affected by the social agency of a stimulus [22]. However, in a recent study, we manipulated participants' perceptions of the movements of a red dot that selected an image as either being controlled by a computer, or another participant's eye movements. We found that accuracy in identifying which image had been selected significantly improved when the movement was perceived as having social agency, i.e. controlled by a participant's eye movements. This was the case both for non-autistic *and* autistic participants. Interestingly, despite displaying better task performance in the eye-movement (social agency) task compared to the computer controlled (non-social agency) task, autistic participants rated this task as being significantly more difficult, indicating that they experience a misplaced lack of confidence in their ability when asked to make judgements in relation to social stimuli [24]. The findings demonstrated that when autistic people believed what they were seeing was being controlled by another human, their ability to judge which item had been selected significantly improved. This suggests that not all autistic adults are necessarily inattentive to other people, as proposed by the social motivation hypothesis, and supports recent research critiquing the social motivation hypothesis [25–27]. Further, these findings demonstrate that even a simple manipulation of the perception of a stimulus as being an independent social agent can be sufficient to drive changes in behaviour for both neurotypical and autistic participants.

Merely implying the presence of another person can lead to behavioural changes. However, the belief that another person is not only present but capable of ‘looking back’ (i.e. observing or judging the participant) can lead to even greater alterations in behaviour for neurotypical participants [23]. This is commonly referred to as the ‘audience effect’, and has been replicated consistently across over a century of research, after first being discussed by Norman Triplett in 1898. Interestingly, in such cases, the observer need not necessarily be present in the same room, or even believed to possess full social agency. For example, in many economic or reputation management tasks the participants may never see or meet the ‘other’ participant (who is often simply a computer algorithm), and yet the implication that another person is both present and observing the participant is found to lead to social facilitation effects. However, audience effects are often more ambiguous within groups of autistic participants [23]. The aforementioned research which argues that autistic participants are less socially motivated than their neurotypical peers is often drawn on as an explanation for why autistic participants may display weaker audience effects [21,23]. In fact, in tasks that require reputation management, autistic participants do not show the same concern for managing their reputation with a social partner as do neurotypical participants [22]. Yet, despite this, autistic participants have still been found to display audience effects and social facilitation on simple cognitive tasks, whereby they show better performance in the physical presence of a social partner [28]. Taken together, the research discussed in this section calls into question the claim that all autistic participants lack sensitivity to social agency. Indeed, the results of these studies suggest that the behaviour of at least a subset of autistic individuals is influenced by a social presence in a similar manner to that of their neurotypical peers. The results of such studies suggest that information on autism that is gathered using stimuli lacking a social presence could be potentially misleading, and may actually limit our insight into how autistic individuals process social stimuli in the real world.

## 4. Interactivity

As outlined previously, while a potential solution to concerns around ecological validity would be to move research directly into the ‘real world’, outside of a controlled laboratory environment, this is often not possible without compromising experimental control. This conundrum has therefore led to the development of innovative paradigms that allow us an approximation of a naturalistic encounter, while also retaining experimental control. An elegant way of demonstrating and quantifying the difference that a naturalistic encounter has is to directly compare behavioural performance between experimental conditions that are identical, save for the key element of social presence. In such cases the stimuli presented to the participants can be identical, for example, if they believe a Skype conversation to be live or a pre-recording and yet participants consistently demonstrate different patterns of behaviour if they believe the stimulus to depict a person in real-time [29]. In a paradigm using the same principle, Gregory *et al*. [30] showed neurotypical participants footage of a confederate sitting in a waiting room, occasionally shifting their location of gaze around the room in a naturalistic way. Participants were either informed that the footage was live, or a recording. In all cases the participants were aware that the confederate would not be able to view them. A question of interest was whether participants would look at the confederates less and be less likely to follow their direction of gaze in the live condition compared to the recorded condition. A clear and striking finding was that participants showed decreased gaze following and visual orienting to confederates in the live condition compared to the recorded condition. Therefore, the participants displayed gaze behaviour typically associated with real world gaze behaviour when in the live conditions, as has also been demonstrated by others [18,31]. In a follow-up study involving autistic adults, the finding that neurotypical adults look more at people if they think they are watching a video compared to a live scene was replicated [32]. However, interestingly, this finding was not observed for autistic people; how much they looked at the people in the scene did not seem to be affected by whether they thought they were viewing a live webcam or a pre-recorded video, indicating that the effect of social presence was not as important to autistic adults. It is possible that while neurotypical people display an inhibition to attend to people when there is a social presence, this inhibition may be weaker in autistic people.

It has been proposed that the difficulties experienced by autistic individuals relate to difficulties in *social interaction* not social observation [12]. Inevitably recorded video-based stimuli remain unaffected by participants' behaviour and are, plainly, unable to respond to social overtures, suggesting that contexts lacking the potential for reciprocal interaction may fail to engage the processes which would be involved in social interactions in everyday life [33]. Such observations have led to the call for ‘second-person’ approaches to investigating social cognition [11,34]. A ‘second-person’ approach involves the participant as an active member of a social interaction [11]. The key argument behind such discussions posits that in order for research to be of clinical relevance, and predictive of real-life behaviour, it is a necessity to increase the interactivity of social cognition paradigms [12]. In support of this claim, a recent study with neurotypical participants found that it was not just the implied presence of a social partner that led to changes in gaze behaviour, but a critical factor was the perceived potential for a social interaction [35]. In this study, all participants were presented with the same 1 min video of a person waiting in a testing laboratory. The video contained one ‘bid for eye-contact’ whereby the person looked directly at the webcam for 4 s. Participants were given one of three descriptions of what was being viewed. They were either told that they were viewing (i) a live webcam stream where interaction was not possible (one-way); (ii) a live webcam stream where interaction was possible (two-way); or (iii) a pre-recorded video. Participants who did not believe the description of what they were viewing were excluded. Participants in the pre-recorded and one-way scenario looked more to the face of their social partner than those in the two-way scenario, particularly, when the confederate made eye contact. It is therefore apparent that even the use of complex, dynamic stimuli where there is no believed potential for social interaction, i.e. no social presence, such as videos, can elicit different patterns of behaviour compared to when there is a believed interactive element, as is the case for real social partners.

Further attempts to ‘bridge the gap’ between naturalistic real-world paradigms and more controlled laboratory-based paradigms have been sought through the use of new technologies, such as virtual reality or augmented reality. Such technology allows the researcher a high degree of experimental control, while still presenting the participant with a highly complex interactive social environment. Studies have shown that neurotypical participants will respond to agents present in augmented reality in a similar way to real social partners [36], and the use of virtual reality paradigms has begun to allow key insights into areas of social cognition, such as emotion processing, for autistic individuals [37]. In a novel study, Jarrold *et al*. [38] generated a public speaking task using virtual reality. In the study, participants were asked to speak within a virtual classroom environment in front of either social avatars, or non-social targets (patterned globes on sticks). Both the neurotypical and autistic participants looked more frequently to the social avatars than non-social targets, and autistic participants did not display reduced social orienting or increased attention disengagement in a baseline condition where they were not required to speak. However, the differences between the groups of participants were most evident in the social condition, whereby when participants were required to speak in front of the social avatars, the autistic participants looked significantly less to some of the social avatars compared to the neurotypical participants. Frequency of looks to the five avatar positions data correctly identified the majority of autistic and neurotypical participants (76% sensitivity; 74% specificity). This paradigm therefore provided key insights into behavioural changes in the presence of ‘social partners’ that assessed aspects of interactive behaviour, while also allowing the experimenter to maintain a high degree of experimental control. Further, the results of this study support previous research which has indicated that individuals with neurodevelopmental conditions can display typical patterns of social attention within settings where they merely observe behaviour, but that differences emerge when interactive elements are introduced [11,12].

While improvements in technology have enabled researchers to more closely approximate social interactions, these paradigms still hold the assumption that behaviours recorded in artificial scenarios are reflective of behaviours within real world encounters [14]. An illustrative example that this is an incorrect assumption was demonstrated by Laidlaw *et al*. [39] who presented neurotypical participants with a waiting room scenario where an experimental confederate was either physically present in the waiting room or presented on a video screen in the waiting room. Participants frequently looked at the video screen confederate but were unlikely to look at the physically present confederate, so unlikely, in fact, that participants were actually more likely to look at an empty chair than when the chair contained a person. Other studies have also demonstrated that neurotypical participants attend less to individuals' faces in real world environments than they do to pre-recorded video stimuli of the same faces on a computer screen [18,31]. Similarly, the oft studied gaze cueing effect [40] seems to be far less pervasive in real life as neurotypical participants appeared reluctant to follow gaze cues of oncoming pedestrians [41]. Therefore, while paradigms such as those discussed in the section above allow us an insight into the importance of social interactions, it is apparent that a further key consideration is how social interactions are influenced when conducted with real, physically present, social partners.

## 5. The face-to-face encounter and autism

Conducting autism and broad autism phenotype research involving real life social encounters has recently become more common [27,42]. The development of structured behavioural tasks and highly accurate, portable behavioural measurement technology (such as physiological measurement, functional near-infrared spectroscopy (fNIRS) and mobile eye tracking) now enable researchers to examine a broad range of social behaviour and associated cortical activity within naturalistic social interactions. In this section, we review studies that aim to improve understanding of how autistic people and those with the broad autism phenotype think and behave during face-to-face encounters grouped by methodological approach.

Structured behavioural tasks have recently provided insight into natural patterns of behaviour. Ochi *et al*. [43] found that clinical diagnostic category (autism/neurotypical) could be predicted with 89% accuracy by focussing on speech features such as pauses, turn-taking and synchrony between an autistic participant and neurotypical researcher during administration of the ADOS-2, indicating that there are predictable, systematic, differences in verbal communication in autistic compared to non-autistic people. However, interestingly, atypicalities in social interaction processes are found to present differently when autistic participants are paired in conversation with another autistic individual. For example, real world social interaction quality in face-to-face dyadic conversations was investigated by Morrison *et al*. [44]. Participants were assigned to one of three dyadic pairing types, these were either autistic–autistic, non-autistic–non-autistic or autistic–non-autistic. Each pair completed a 5 min unstructured conversation following which each individual rated the quality of the interaction and their impressions of their partner. Autistic participants trended towards an interaction preference with other autistic adults and reported disclosing more about themselves to autistic compared to typically developing partners, indicating that social affiliation may increase for autistic adults when partnered with other autistic people. Using a similar dyadic pairings set-up, Crompton *et al*. [45] examined information transfer between autistic adults, non-autistic adults and mixed autistic–non-autistic pairings whereby initial participants were told a story which they recounted to a second participant, who recounted the story to a third participant and so on, along a ‘diffusion chain’ of eight participants. Participants were situated in separate rooms throughout the study save for when participating in the diffusion chain. Findings were that autistic people were just as able to effectively share information between one another when recounting stories in face-to-face settings as non-autistic people were, challenging the commonly held view that autistic people lack the skills to interact successfully. It was only in mixed autistic–non-autistic pairings where information transfer broke down. Via such carefully controlled, face-to-face structured interaction scenarios important information on the true nature of social interaction processes can be revealed. Further, structured behavioural tasks can also provide insight into isolated aspects of naturally complex behaviour. For example, we recently conducted a face-to-face gaze following task where participants were asked to point to the exact location of an experimenter's gaze across a series of carefully paced and timed trials. We observed that both autistic and non-autistic adults were able to effectively follow an experimenter's gaze direction following both long looks and brief glances, though performance of autistic individuals was overall less accurate than neurotypicals [46]. It is possible that the suggestion that some autistic individuals find direct eye-contact aversive [47,48] may lead to reduced experience with direct eye-contact, and therefore fewer opportunities to practice gaze following in real-life situations. This may contribute to reduced accuracy with the skill of gaze following overall. This may also be why we do not see reduced sensitivity to social agency in autistic people when tasks do not involve the physical presence of eye stimuli [24] as the aversive stimulus, a pair of eyes, is not present.

Using physiological measurement it has recently been found that direct gaze from a real person leads to changes in skin conductance response for neurotypical participants compared to conditions where there is no direct gaze [49]. However, if participants can see another person but believe that a semi-silvered mirror results in the social partner being unable to see them, participants show reduced skin conductance response to direct gaze, indicating increased arousal only when participants believe they are being observed [50]. This technique has the potential to provide insights into levels of arousal associated with different aspects of social encounters in autistic people which may, otherwise, be difficult to assess. For example, Tanaka & Sung [48] discuss that avoiding attending to the eyes is an adaptive strategy in autistic individuals, as direct gaze is shown to elicit increased skin conductance responses associated with the perception of threatening behaviour. However, they discuss that while this is adaptive in terms of threat avoidance, it can lead to challenges for autistic individuals in reading emotions and intentions from other people. The development of fNIRS technology now provides the opportunity to assess cortical activation during social encounters. Suda *et al*. [51] used fNIRS to assess the relationship between prefrontal cortex/superior temporal sulcus activations and autistic traits during face-to-face conversations in neurotypical adults. These brain regions are critically implicated in processing social stimuli in neurotypical participants. Participants were either required to talk about food with three unacquainted male researchers, or participants were asked to repeat meaningless syllables such as ‘a’, ‘ka’, ‘sa’, ‘ta’ and ‘na’. The fNIRS results revealed higher activation in the prefrontal cortex and the superior temporal sulcus (STS) during face-to-face conversations than the syllable repetitions, but importantly a significant negative correlation between participants' autistic traits and left STS activation in the face-to-face conversations. This suggests that the increased presence of autistic traits led to less activation in brain regions critical for processing social stimuli. Another example study using fNIRS during genuine social encounters was conducted by Su *et al*. [52], who asked autistic children to take part in a face-to-face interpersonal synchrony task. They found that the autistic children displayed hypoactivation of brain regions related to imitation and interpersonal synchrony (e.g. middle inferior frontal gyrus and middle superior temporal gyrus), but heightened activation in brain regions associated with motor planning (e.g. inferior parietal lobule), which they suggest could serve as a potential biomarker for autism.

The other main technique for assessing behaviour on a moment by moment basis during genuine social encounters is mobile eye-tracking. As would be expected, such studies have demonstrated that autistic individuals have a general tendency to avoid looking to the eye region when faced with a real-time social partner [2,53,54], with this effect being stronger when a social partner makes direct eye-contact with the autistic participant compared to when the social partner averts gaze [55]. In this study, we also demonstrated that autistic people displayed effective social modulation when switching between listening and speaking phases of conversations, in a similar manner to neurotypical people. Overall, the studies described demonstrate the types of insights that can be gained from structured face-to-face tasks revealing the aspects of social stimuli that can be found aversive by autistic people and the strategies that are naturally used by autistic people to counteract the impact of these during genuine social encounters.

## 6. Generalizability of findings from computer-based interactions to real-life encounters

Considering how the findings of computer-based studies in relation to social attention scale up to genuine real-life social interactions, it may seem logical to predict that any differences in behaviour between autistic and non-autistic people observed in computer-based tasks would only be amplified when assessed in real-life scenarios. However, a range of recent studies have demonstrated that this is, in fact, not the case. Cañigueral *et al*. [56] engineered a one-to-one social interaction scenario whereby a conversation with an experimenter was either presented to participants via a pre-recorded video, by a live online interaction or via a face-to-face encounter where only the experimenter's face and upper torso could be seen. The researchers ensured that the physical appearance of the experimenter in each of the three conditions was as similar to possible. Eye gaze and facial motion patterns in autistic participants were found to be overall similar to the neurotypical participants across all conditions. This study, therefore, provides evidence that differences in behaviour between autistic and non-autistic people are no more evident during a face-to-face encounter than when engaged in a task that does not involve a social presence.

A study by Grossman *et al*. [57] observed reduced looks to faces in autistic participants compared to non-autistic participants in a screen-based task but not a live interaction. Again, a demonstration that differences were not more evident when a social presence was involved. We also found that autistic traits were only associated with less looking at a partner's face when the partner appears on a video, but not in real life [58]. Similarly, we recently observed that increased autistic traits were not correlated with reduced looking at the social partner in general, or their face more specifically, during a structured face-to-face conversation task. However, we did observe that individuals who were high in autistic traits exhibited reduced visual exploration overall during face-to-face interactions, indicating that precise analysis of behaviour can reveal somewhat subtle differences that may have profound downstream effects [59]. A potential explanation for why autistic traits do not inevitably lead to strikingly atypical patterns of social attention in live situations is that in live interaction scenarios many autistic people, and those high in autistic traits, are likely to engage in masking behaviour, whereby learned, practised patterns of behaviour in order to ‘fit in’ or appear more neurotypical are produced [60]. For example, people are often told to ‘make eye contact during conversations'. However, there is far less societal awareness of more nuanced social attention rules that neurotypicals generally abide by without being aware that they are doing so, meaning that some of these more subtle rules are less likely to be instructed or spontaneously learned. Similarly, opportunities, and the necessity, to practice social attention behaviour may be far less extensive when observing screen-based interactions, hence making easily observable differences between autistic and non-autistic patterns of social attention more likely. In any case, whatever the exact reasons for the different patterns of behaviour in real social encounters compared to computer-based tasks, that these differences exist demonstrates the importance of considering behaviour within genuine social encounters else our understanding of the behaviours we claim to be most interested in will be incomplete.

## 7. Identity of the social partner matters

It has been suggested for some time that the identity of the social partner matters when considering social mechanisms and processes. However, in much experimental research often partners are merely presented as anonymous prompts whereby the genuine interaction aspect brought by the social partner is not considered further. However, as explained by Kuhlen & Brennan [61], it is important to consider how a confederate may influence a task compared to having only naive participants, i.e. more genuine social partners, as part of a task. It must be considered whether a confederate can fulfil the role of a conversational partner without unduly influencing the nature of the conversation and thus the conclusions drawn. This discussion is particularly pertinent to autism research given the prominent, influential theory of the double empathy problem [62]. The double empathy problem outlines the existence of a two-way communication challenge in social expression and understanding between autistics and neurotypicals that present barriers for cross-diagnostic interaction and connection. The idea that neurotypicals struggle with interpreting the social cues of autistics just as much as autistics struggle with interpreting the social cues of neurotypicals has received clear empirical support in recent years (e.g. [44,45,63,64]). It is only when autistic–non-autistic pairings are analysed that the quality of interactions reduces [45]. It is therefore crucial to consider the identity of the social partner when attempting to interpret and draw inferences from behavioural studies on social interaction involving autistic people.

## 8. Moving beyond social attention: the effect of social presence on other areas of social cognition

The current landscape of research focussing on ‘real’ social interactions is one of new, exciting and innovative methodological techniques and paradigms. Yet, at the current time the focus of such research is limited to relatively specific research areas such as social attention [14,16], joint action [65] and conversation [44]. As highlighted throughout this review, studies across these areas have provided an understanding of how critical it is to study social behaviour within social contexts and a key understanding now emerging is that interactive processes are not just merely a context in which behaviour happens. Instead, they are a critical component of human cognitive processing and can drive and instigate cognitive mechanisms in their own right [12,66]. In the light of these new findings, one of the goals of future research should be to focus on investigating if social presence plays as key a role in other areas of social cognition as it clearly does in those researched so far.

Social cognition covers a broad range of cognitive abilities that enable us to process and respond to the social world in which we exist [67]. However, in line with several other areas of research, paradigms used throughout these areas have traditionally given little consideration to the social complexity of the stimuli used in each task and the true impact of social presence on the findings in this field are only just beginning to be understood. In the field of perspective taking research, task stimuli can vary between pictorial representations of people [68] to live tasks presented by a confederate [69]. Similarly, in emotion recognition research, the task stimuli can vary from static, regimented presentations of actors such as those outlined by Eckman [70] to more complex emotional stimuli presented via video presentations or, relatively recently, via virtual reality avatars [37,71]. Yet, despite the clear differences in social complexity in the stimuli used in these paradigms, it is not yet understood if these differences lead to empirical alterations of behaviour. This is a critical consideration as these research areas encompass skills and behaviours which are associated with differences or difficulties for autistic individuals (DSM-5, [1]), and are often drawn on to support our understanding of autistic behaviours and traits. This is of concern as we do not have a clear understanding of what impact social presence has on these social behaviours, and hence if the behaviours recorded via these paradigms are reflective of real life functioning.

An area of social cognition research that we have chosen to focus on in our own work is that of ‘theory of mind’ research. Similarly to both emotion recognition and action recognition research, paradigms used in the field of theory of mind research can vary greatly in terms of their social complexity, and the impact of real people on the behaviours of interest is not often considered [72]. There is therefore a scarcity of research investigating how theory of mind processes behave in ‘real life’. This is, perhaps, surprising when one considers how many of our daily interactions depend upon being able to implicitly infer and recognize the motivations and intentions of our social partners. If social attention demonstrates that our attention is drawn to social stimuli and allows us to process ‘what’ we are looking at, theory of mind abilities deepen the process started by social attention and allow us insights into the ‘why’ of other people's behaviour. Therefore, while theory of mind research represents its own field within social cognition research, it is also implicated across a far broader range of behaviours and processes and is argued to underpin other critical social skills by allowing us insights and guiding our reactions to the emotions [73], perspectives [74] and actions [75] of our social partners. A lack of clear understanding of how this ability behaves *in situ* is further surprising when we consider how difficulties in this particular skill were once considered a hallmark of autism, and broadly accepted as a theoretical basis to explain the social and communicative differences associated with an autism diagnosis [76]. Such conclusions were drawn from socially isolated laboratory-based paradigms, conducted with simplistic stimuli such as cartoon sketches [77] and are therefore potentially problematic when we consider what we are now beginning to understand regarding the impact of social presence on other areas of social cognition. For these reasons, we investigated whether social presence is a factor that can influence conclusions relating to theory of mind research [72]. Across two studies we presented participants with a commonly used theory of mind paradigm known as a ‘false belief’ task. Critically, participants watched the task in two conditions. In one condition the task was acted out live in front of the participant by task confederates, in the other condition the participant watched recorded videos of the same confederates acting out the same task. We paired this task with two commonly used behavioural measures to measure task performance, eye tracking or a direct response from the participant; such as a key press or finger point. Across both studies, participants' eye movements were found to be significantly influenced by whether the task was presented live or on a computer screen, such that the participants’ eye movements were more likely to demonstrate behaviour associated with accurate mentalizing (directing their attention to the location they believed a confederate would search) when the task was completed with live confederates. This finding was later replicated in a follow-up study with autistic adults and age, gender and non-verbal IQ-matched controls (Morgan *et al*. [78])^1^. The results of these studies therefore provide evidence that a social presence can lead to quantifiable changes in behaviour on certain behavioural measures (such as eye tracking) when completing theory of mind tasks for both autistic and non-autistic adults. Studies that attempt to draw conclusions about mentalizing ability without real-time protagonists are therefore likely to miss out on crucial aspects of this ability.

## 9. Conclusion and future directions

The aim of this review was to consider whether and how the presence of a social partner i.e. ‘social presence’ affects social attention, social information processing and social interaction behaviour in both autistic and non-autistic people. This review has provided clear evidence that social presence does indeed strongly influence all of these factors in both autistic and non-autistic people. Relatedly, we find participants are not only sensitive to social presence across a range of different scenarios but that they are also acutely sensitive to the potential for interactivity. Further, they also adjust their behaviour in response to even minor perceptual changes in the social nature of a stimulus, in the absence of social presence, such as whether the stimulus has social agency. The identity of the social partner matters and the effects of social presence go beyond social attention into other social cognition domains, such as theory of mind. It is therefore imperative not to overlook the potential influence of these factors when designing future studies, making inferences and generating theory.

Throughout this review, we have demonstrated that not only is social presence a topic of concern for social cognition research, but that over the last decade we have seen many novel, innovative and technologically cutting edge paradigms and techniques that have helped to take account of the influence of social presence as a potentially important factor. The result is paradigms that involve stimuli being able to ‘look back’ at the participant, whereby the participant can be observed or judged, and the participant being able to ‘look back’ at the stimulus. Such paradigms have demonstrated that computer-based, disembodied paradigms are unlikely to provide a complete picture of real world behaviour, and that ecologically valid studies have an important contribution to make. This is critically important when we consider the implications on understanding conditions such as autism. Many theories and interventions that have been developed with a view to improving understanding of autistic social interaction processes, or social cognition more broadly, have been informed by computer-based, socially isolated research. As outlined in this review, recent research using more ecologically valid paradigms, containing elements of interactivity, do not support the hypotheses that all autistic individuals lack social motivation, that all autistic individuals lack sensitivity to the presence of social partners, or that all autistic individuals do not attend to social stimuli. Instead, the research discussed throughout this review suggests that these previously held beliefs are erroneous, and that autistic individuals' perceptions and interactions with stimuli that possess social agency are far more nuanced than previously proposed. Indeed, we have offered evidence here that rather than lacking a ‘sensitivity’ to social agency and social presence, instead many autistic people may struggle with finding certain elements to do with these stimuli, such as direct eye-contact, aversive. It is also clear that autistic people often struggle with social confidence and social understanding owing to being autistic in a majority neurotypical world, this also probably contributes to many observed differences. A lack of social confidence and social understanding could well be societally driven, whereby a lack of understanding from non-autistic people in terms of preferred autistic social interaction styles could underlie many difficulties experienced, and autistic–autistic interactions can be more successful than mixed autistic–non-autistic interactions.

The research discussed throughout this review leads to new and exciting directions of future research, both for neurotypical and autistic adults. In particular, while we are developing a clearer understanding of *how* social presence influences participants' behaviour, there is still much future research necessary in order to understand *why* it influences participants’ behaviour. That is, there is still much debate surrounding the exact underlying mechanisms that produce the behavioural differences observed between socially isolated paradigms, and those conducted with at least the perception of the presence of another person [79]. Previous attempts have alluded to the importance of social norms in influencing real world behaviour [58], the engagement of additional mentalizing processes in the presence of real people [24,34], the potential for social interaction [12] or the engagement of reputation management processes [80] as critical underlying factors. Most recently, the social signalling framework lays out a model in order to help quantify and test the engagement of which neurocognitive mechanisms are engaged in social interaction paradigms. This framework is based on the understanding that it is both the ability to send and receive social signals to a social partner which helps to instigate these changes in behaviour [79]. This remains a highly active and evolving field of study, and our understanding of these mechanisms is only likely to increase in the coming years. Similarly while the research discussed in this review offers positive and necessary future directions in autism research, it must be acknowledged that many gaps remain to be addressed, not least that many of the findings discussed here are drawn from a subset of the population of autistic adults who do not have co-occurring intellectual disability.

Overall, the evidence presented in this review suggests that our current understanding of autistic social interaction processing is fragmented and, in some ways, flawed. Many previous assumptions regarding social cognition in autism must be revisited to determine if they are an accurate depiction of social engagement in everyday life. It is therefore evident that in the future, more face-to-face studies are required, using naturalistic paradigms in order to develop a framework of understanding with the aim of being genuinely useful to autistic people.

## Data Availability

This article has no additional data.

## Footnotes

1 Data and analysis syntax are available on the Open Science Framework: https://osf.io/t82yh/.
